# Evaluation of Mothers’ Perceptions of a Technology-Based Supportive Educational Parenting Program (Part 2): Qualitative Study

**DOI:** 10.2196/11065

**Published:** 2019-02-13

**Authors:** Shefaly Shorey, Esperanza Debby Ng

**Affiliations:** 1 National University of Singapore Singapore Singapore

**Keywords:** education, mothers, parenting, technology

## Abstract

**Background:**

Transitioning into parenthood can be stressful as parents struggle to cope with new parenting responsibilities. Although perinatal care in hospitals aims to improve parental outcomes, there is a general consensus that it is suboptimal and insufficient. Therefore, many studies have designed intervention methods to supplement support for parents during this stressful period. However, studies often focus on parental outcomes as indicators of their interventions’ success and effectiveness. Studies evaluating participants’ experiences and feedback are limited.

**Objective:**

This study aimed to examine the experiences and perceptions of participants who participated in a supportive education parenting program intervention study.

**Methods:**

A qualitative semistructured interview was conducted with 16 mothers (6 control and 10 intervention) from a randomized controlled trial. The supportive education parenting program received by the intervention group included 2 phone-based perinatal educational sessions, a phone-based educational session after childbirth, and a 1-month postpartum access to a mobile health app. The interviews were approximately 30- to 60-min long, audiotaped and transcribed verbatim, and analyzed using thematic analysis. Study findings were reported according to the Consolidated Criteria for Reporting Qualitative Research checklist.

**Results:**

The 3 main themes evaluating mothers’ experiences and perceptions were generated: (1) changed perspective toward parenthood, (2) journey from pregnancy to after birth, and (3) a way forward. Mothers from the intervention group mostly had good perinatal experiences with sufficient support received, which elevated their emotional well-being and increased parenting involvement. Mothers in the control group, although satisfied with the hospital care received, were more stressed and shared a need for professional advice and extra support. Apart from technical enhancements, mothers also requested extended social support during early pregnancy up to 1 year postpartum, taking into consideration Asian cultural practices.

**Conclusions:**

Mothers who received the intervention were overall satisfied with the support provided by the technology-based supportive educational parenting program. The success of the educational program in this study highlights the need to supplement standard care in hospitals with technology-based educational programs. Future research should include fathers’ perceptions to attain an in-depth understanding of overall participants’ experiences and needs in the future development of supportive and educational programs.

## Introduction

### Background

The birth of a child is typically a joyous occasion, but it can elicit an amalgamation of emotions from parents, ranging from elation to anxiety. Transitioning into parenthood can be stressful as parents often feel overwhelmed while struggling to achieve a new balance between their work lives and infant care [1,2]. This is especially challenging for mothers who are the sole caregivers and lack social support in the postpartum period [3,4]. Mothers have to constantly monitor their babies’ health, engage in baby care tasks, breastfeed, and experience varied sleep patterns, which cause high stress levels and increase their susceptibility to postpartum affective disorders such as postpartum anxiety and postpartum depression [5-7].

To this day, specific causes of postpartum psychiatric disorders remain unknown, although many studies suggest a multifactorial etiology [3,6,8]. However, certain protective factors such as social support received [9] and parental self-efficacy [10,11] are known to elevate maternal moods and keep postpartum psychiatric disorders at bay [10-12]. Maternal moods also adversely affect paternal psychological well-being [13], which subsequently disrupts parent-child bonding [14-16] and jeopardizes marital relationships [15] as negative emotions resonate off each other, creating a complex snowballing cycle. The resulting poor family dynamics and unconducive home environment, in turn, affect the child’s physical, behavioral, and cognitive development [16,17].

To avoid negative social consequences inflicted by high maternal morbidity and poor child development, hospitals have implemented perinatal classes to prepare mothers for parenthood. However, there is a lack of continuity of care after hospital discharge [18], and the current perinatal care provided by hospitals is reportedly suboptimal and insufficient in meeting mothers’ needs [19].

Postnatal follow-ups often only focus on breastfeeding and the physical well-being of mothers, often neglecting mothers’ psychological and emotional well-being [20]. Moreover, in 1 study by Ong et al [19], because of short hospital stays, unawareness, lack of time, or financial constraints, very few mothers attended perinatal educational classes by hospitals. This can result in a lack of preparedness and poor parenting self-efficacy (PSE), which not only affect parental bonding and maternal emotional well-being but may also affect future childbearing decisions [21]. However, the Asian practice of a confinement period, which typically lasts for a month and mainly involves a confinement lady or the new mother’s own mother or mother-in-law taking care of the special dietary needs of the new mother and infant care tasks, was shown to provide instrumental support and improve maternal outcomes during the postpartum period [22].

In the effort to help parents tide over challenging times in the perinatal period, many preventive and treatment programs were designed to improve parental outcomes and reduce postpartum affective disorders, which were mostly successful [10,23-25]. Successful intervention methods have included home visits [26], telephone call follow-ups [27], and face-to-face educational programs [10,28], but they required significant resource costs because of practitioner involvement. An ideal solution is to harness widely available and advanced technology to provide sustainable and cost-effective health care [29]. However, there is a lack of available literature on multimodal technology-based interventions administered during the perinatal period, with most available studies adopting a single modal approach [23,24]. The majority of existing literature [9,30,31] tends to focus on at-risk parents, often neglecting healthy groups of parents who are also going through this stressful postpartum period. Therefore, there is a need for a universal intervention that is also applicable to the healthy population. In addition, most randomized controlled trials (RCTs) only focused on the quantitative analysis of parental outcomes; hence, the qualitative evaluations of participants’ perceptions of such perinatal technology-based interventions are limited. Considering that similar interventions may have different results depending on the setting and the population group involved, it is important to incorporate detailed insights on users’ experiences and feedback on the intervention when gauging its success and effectiveness on parental outcomes [32]. A qualitative analysis also provides descriptive and exploratory insights that can better capture complex social needs and allow intervention methods to be tailored according to the population’s needs [33]. Process-oriented studies evaluating parenting programs have increased substantially, but the programs are often home-based or face-to-face [34-38], which differ in administration and intervention components compared with technology-based programs. The heterogeneity of the studies in terms of findings or themes identified [39] and interview sample population (eg, fathers [40], immigrants [35], health care professionals [37], and low-income mothers [38]), warrants a need for a qualitative study specific to the current educational program.

### Aim of the Study

Therefore, the aim of this study was to explore the experiences and perceptions of participants in a technology-based supportive educational parenting program (SEPP). The perinatal experiences of the control group were included to show the contrast in experiences from those who received the intervention.

## Methods

### Design and Setting

This study adopted a descriptive qualitative study design. It is a follow-up of an RCT that examined the effectiveness of a technology-based SEPP on parental outcomes during the perinatal period [41]. The original study took place in a tertiary hospital in Singapore, where a convenience sample of 118 couples was recruited. Participants in the control group only received routine hospital perinatal care, whereas those in the intervention group received additional phone-based educational sessions during the perinatal period and access to a mobile health (mHealth) app for 1 month post delivery. The 2 antenatal and postnatal phone-based education sessions were administered by a professionally trained research assistant. The details of each SEPP component can be found in the study protocol [41]. Despite both parents being involved in the initial trial [41], the unavailability of fathers because of their work commitments limited the interviewees to mothers only. Mothers from the control group were also included in this study to provide a comparison of perinatal experiences and further insight into unmet maternal needs. Before the intervention study, participants were already informed of this optional extended qualitative arm of the study and that they would be further reimbursed for participation.

### Recruitment

Recruitment took place 1 month postpartum (immediately after the completion of the intervention). Participants in this study were a subset of mothers involved in the initial intervention study [41]. Convenience sampling was conducted through the blasting of messages to all 118 participants (from both intervention and control groups) until data saturation was reached. Overall, 30 mothers volunteered to be interviewed, but data saturation was reached at the 14th participant, when no new findings emerged. Moreover, 2 additional interviews were performed to confirm the findings, resulting in a total of 16 participants (6 from the control group and 10 from the intervention group). The remaining 14 mothers agreed to withdraw from the study. The mothers were notified of the estimated length of the interview (approximately 30-60 min) and that all interviews would be audio-recorded for research purposes only.

### Data Collection

A research assistant trained in qualitative interviewing techniques conducted the face-to-face interviews with the mothers at a time and location of each mother’s convenience, which was typically during their postnatal follow-ups in the hospital. A semistructured interview guide was adopted with probing questions to attain a more comprehensive view of the effectiveness of the SEPP. The interview guides for both groups are presented in Textboxes 1 and 2. The interviews lasted approximately 30 min and were subsequently transcribed verbatim. Field notes were taken during the interviews to note nonverbal cues that were used to supplement the transcript.

**Qualitative evaluation semistructured guide for the control group.**

Probing question

1. How did you prepare yourself during the pregnancy? Did you seek any support?
2. How did you bond with your baby? Did you think it was effective?
3. How did you provide newborn care? Did you find it efficient?
4. Did you seek any social support regarding newborn care or postdelivery care?
5. How did you feel emotionally during the pregnancy and post delivery?
6. How did you gain knowledge on self-care and newborn care during your pregnancy or post delivery?
7. Did you think any nurse’s/midwife’s expert advice will be beneficial during the pregnancy or post delivery?
8. Do you think it is worthwhile to spend extra time receiving educational programs provided by the hospital? Or any peer support groups?
9. How did you find the perinatal care provided to you by the hospital throughout your pregnancy, during labor, and after delivery?
10. Do you have any suggestions for the improvement of care or support for example during pregnancy, labor, and after delivery, provided to you as a parent?

**Qualitative evaluation semistructured guide for the intervention group.**

Probing question

1. How did you feel participating in this research study?
2. Was this supportive educational parenting program helpful?
3. Did you find the supportive educational parenting program useful in improving your bond with your newborn?
4. Did you find the supportive educational parenting program useful in improving your self-efficacy in newborn care?
5. Did you find the supportive educational parenting program useful in improving your social support seeking behavior?
6. Did you find the supportive educational parenting program useful in improving your mood and decreasing your negative emotions?
7. Did you find the supportive educational parenting program useful in improving your knowledge about self-care and newborn care post delivery?
8. Did you find the nurse’s/midwife’s expert advice beneficial?
9. Do you think it was worthwhile spending extra time to receive this supportive educational parenting program intervention?
10. What were the strengths and weaknesses of the intervention?
11. How did you find the perinatal care provided to you by the hospital?
12. Do you have any suggestion for the improvement of perinatal support provided to you as a parent?

### Data Analysis

Thematic analysis was conducted by 2 authors independently according to Braun and Clark’s [42] 6 phases of analysis. The authors read the 16 transcribed interviews multiple times to familiarize themselves with the data. A manual color-coding method was employed to highlight different concepts and generate the initial codes. Related codes from all excerpts were collated to generate subthemes and overarching themes, which were reviewed comprehensively for homogeneity by both authors. Field notes were also constantly referred to as supplementary materials. Any discrepancies were discussed and clarified between the 2 authors until consensus was achieved. Upon further discussion, prominent themes constituting frequently reported overlapping data were selected from the authors’ independent analysis, renamed, and included in the final analysis.

### Ethical Considerations

Ethics approval was obtained from the National Health Group Domain Specific Review Board (Reference Number: NHG DSRB: 2016/00651) of the participating hospital. Relevant information about the research study was comprehensively explained to the participants, and the participants were informed of their rights to withdraw at any time during the study. After which, a written informed consent was obtained from each participant. Participation in this research was strictly voluntary and confidentiality was adhered to.

## Results

Study findings were reported according to the Consolidated Criteria for Reporting Qualitative Research checklist [43].

### Participant Characteristics

A total of 16 mothers were interviewed (6 control and 10 intervention). They had an age range of 23 to 41 years. The majority of the mothers were Chinese (n=9), followed by Malay (n=3), Indian (n=3), and Pakistani (n=1) (Table 1).

### Themes and Subthemes

**Figure 1:**
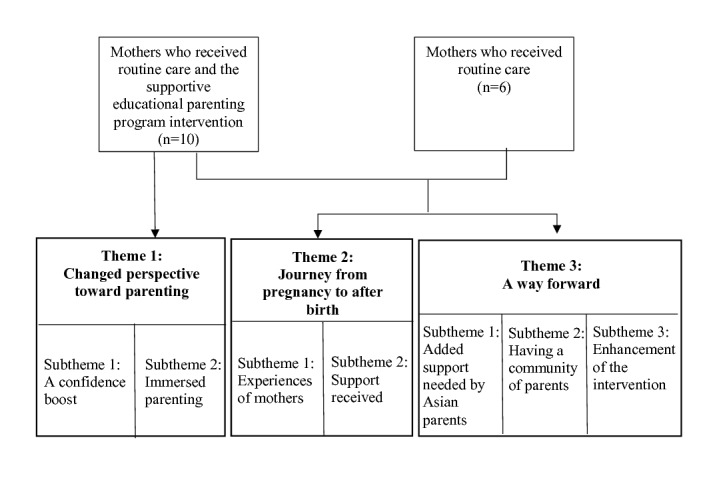
Themes and subthemes.

Overall, 3 major themes were identified (Figure 1): (1) changed perspective toward parenthood, (2) journey from pregnancy to after birth, and (3) a way forward.

**Table 1:** Description of the interviewed mothers (n=16).

ID	Group	Age (years)	Ethnicity	Employment	Antenatal class	Type of delivery	Duration of maternity leave	Confinement period	Child birth order
C1	Control	31	Indian	Employed	No	Assisted	>12 weeks	No	First
C2	Control	27	Malay	Employed	Yes	NVD^a^	>12 weeks	Yes	Second
C3	Control	41	Malay	Unemployed	No	NVD	NS^b^	Yes	Second
C4	Control	34	Chinese	Employed	No	NVD	>12 weeks	Yes	First
C5	Control	34	Chinese	Employed	No	NVD	NS	Yes	Second
C6	Control	30	Chinese	Employed	Yes	C-sect^c^	≤12 weeks	Yes	Second
T1	Intervention	33	Chinese	Employed	No	C-sect	>12 weeks	Yes	First
T2	Intervention	34	Indian	Employed	No	NVD	≤12 weeks	No	First
T3	Intervention	28	Chinese	Employed	No	NVD	≤12 weeks	Yes	First
T4	Intervention	34	Chinese	Employed	No	NVD	>12 weeks	Yes	≥ third
T5	Intervention	23	Pakistani	Unemployed	Yes	C-sect	NS	Yes	First
T6	Intervention	25	Chinese	Unemployed	No	NVD	>12 weeks	Yes	First
T7	Intervention	28	Chinese	Unemployed	No	NVD	NS	Yes	First
T8	Intervention	29	Indian	Employed	No	C-sect	≤12 weeks	No	First
T9	Intervention	32	Malay	Employed	No	NVD	>12 weeks	Yes	≥ third
T10	Intervention	30	Chinese	Employed	Yes	NVD	≤12 weeks	Yes	First

^a^NVD: normal vaginal delivery.

^b^NS: not stated.

^c^C-sect: cesarean section.

### Theme 1: Changed Perspective Toward Parenthood

Overall, mothers who received the intervention had improved emotional well-being and confidence levels in handling baby care tasks. This, in turn, led to more involved parenting and effective parental bonding, which encouraged their partners’ active involvement as well.

#### Subtheme 1: A Confidence Boost

The majority of the mothers from the intervention group felt more relaxed, encouraged to seek help, and confident in taking care of their babies. One mother shared how the postnatal education phone call helped her to manage her emotions better and built her confidence:

The starting period when I was having difficulties, the phone call supported me a lot...to understand my own emotions because I was depressed at the start, and I was restless not having enough sleep...Also, I got some confidence in understanding my baby...

T11

A different mother shared how the mHealth app improved her baby care skills, which increased her confidence:

Through this application, there was a page on how we should take care of the baby’s bathing and I have also seen a video...Like, from 0%, at least, I have improved to 70%. I can confidently say that I can take care of the baby, but I am still learning...

T8

In addition, the majority of the mothers stated how the forum component of the app encouraged help-seeking behaviors and motivated them. According to 1 mother:

Sometimes, mothers don’t dare to ask questions...So, if you ask through such a platform, no one else knows that you ask questions and you still get the answers you need.

T4

Apart from the given anonymity and the reduced stigma, 2 mothers also shared how the forum:

encourages you, like you don’t have to be shy to ask.

T9

And that:

it makes you more willing to speak up...they are also facing the same issues and, then, they are overcoming them well, which gives you more confidence and motivation to persevere.

T3

#### Subtheme 2: Immersed Parenting

On receiving this intervention, most mothers reported improved bonds with their babies. Moreover, 1 mother shared that the self-care tips and infant care information available on the app helped with maternal bonding:

For the first few weeks, I was not able to have a proper [maternal] attachment with the baby, but I read through how I have to take care of myself after my C-section, and, then, I had bonding with my baby and...I read through the positions on how to hold my baby...So, it helped me bond with my baby...

T8

Another mother shared that the information on the app allowed her to understand her baby better, which enhanced her bonding:

I found [out] how to take care of the newborn in terms of basic needs and stuff, making the baby more pleasant and happy. So, when he is happy, it is easier to bond...

T1

She also added:

Because of the video, my husband is more knowledgeable about breastfeeding...So, it is like a guidebook to him too...He is more supportive towards breastfeeding now.

T1

A first-time mother and an experienced mother also gave feedback on increased paternal involvement because of educational information and reminders provided by the app:

It helped my husband to bond with my baby...He hasn’t carried any baby in his lifetime...So, the application...helped him a lot.

T8

Because she is already the third child, so bonding time with the father is not as much as with me...So, I guess with this app, it reminds us [that] the father needs to still be involved because, I mean, the father honestly spent a lot of time dealing with the other two [kids]. Sometimes, I will take over and, then, he comes around.

T4

### Theme 2: Journey From Pregnancy to After Birth

This theme encapsulates the journeys and experiences of mothers during their last trimester to 1 month postpartum, including the support they received during this period. Overall, mothers from both groups (intervention and control) had positive pregnancy experiences; however, mothers in the control group were more stressed than those in the intervention group after delivery. Most mothers from both groups were also satisfied with the care and support received, but mothers from the control group highlighted the need for professional advice.

#### Subtheme 1: Experiences of Mothers

During pregnancy, a first-time mother from the control group reported being emotionally scared because she did not know what to expect. In contrast, first-time mothers in the intervention group felt good and excited. One of them shared:

My mood was very good...as they [phone call education] told me what to expect...I was excited to see my baby.

T10

After delivery, although some mothers from the control group reported that they were managing well, they also frequently used the words *stressed*, *anxious*, and *challenging*. A mother who had undergone a cesarean section shared her postnatal experiences:

We were quite helpless sometimes because we are new parents...I felt incompetent...It was more on anxiety and, sometimes, anger at myself for not being able to recover in time...I didn’t really know whether, with my wound [still recovering], I can actually care for her in a right, responsible way.

C4

On the contrary, mothers from the intervention group reported feeling less anxious:

Actually, I feel more at ease, not worried. I am not that worried that I am feeding wrongly and stuff...as my queries have been answered in a timely manner.

T2

#### Subtheme 2: Support Received

Mothers from both groups were overall satisfied with the hospital care received and the social support received from their loved ones:

I think the perinatal care itself...was very good according to me. Even after the delivery, we did get visits to see a better doctor and so on. So, I didn’t feel that anything was lacking, so I was quite happy with the service that I got overall.

C5

I am really pleased to be here. For once, they responded very quickly to the request...and every nurse was very willing to do things for you...and they are very nice when approaching you and teaching you...so I feel like I am in good hands.

T4

However, despite the availability of external platforms (ie, the internet, Facebook groups, books, or confinement ladies) on infant and self-care information, several mothers from the control group still shared the need for advice and emotional support from a health care professional whom they could trust. One mother justified her need for added professional advice despite having support from a confinement lady, an experienced family member, and the internet:

If we have other information, like more on scientific ones...I think it is more helpful...would be better if we have somebody else...an expert...to tell us exactly what is the correct one because...whatever we have, we actually learnt from the confinement lady, so we are not sure whether that is really, like, the correct thing to do, la...and if I tell my sister-in-law the situation, she might come across [different problems] because every baby is different, so it [her solution] might not be applicable to my baby...Sometimes, we share...but it might not actually be the solution for the problem...Sometimes, it is reassuring to know that it comes from a nurse or midwife rather than any Google or website.

C4

On the other hand, mothers who received the intervention felt more prepared, reassured, and encouraged by the professional advice and existing external support they got:

It definitely helps us to get answers [from the midwife], not spend time worrying, and, then, you Google and, sometimes, we are even more scared. Because we got answers from professionals and they also always replied in a very positive tone, which really helped us to be very confident and just not worry too much...

T4

An experienced mother shared how the app served as a *refresher course*:

The gap with my third one is quite far, about 5 years. I actually forgot what I must do. What is good for the baby? When somebody replies to you, “Oh, okay, I know this.” It [the app] is something like a refresher course.

T9

### Theme 3: A Way Forward

This theme explores the feedback and opinions from mothers in both groups, which primarily acknowledges the need for additional social support in the Asian context, the importance of being part of a community of new parents, and suggested improvements to this intervention study.

#### Subtheme 1: Added Support Needed by Asian Parents

Despite the Asian culture being known for its collectivistic culture and its strong emphasis on family-oriented values, certain cultural taboos restrict help-seeking behaviors during early pregnancy. According to a mother:

For the Chinese, the first month of the first trimester is a hush-hush kind of thing because we are a bit pantang [superstitious], you know, they [are] afraid that if you tell everybody that you are pregnant, then...[something bad will happen].

C4

In addition, although confinement practices were thought to provide more support to mothers during the first month postpartum, 1 mother confessed that having a confinement lady only delayed parenting stress by 1 month. Another mother shared the need for more support, especially after 1 month postpartum:

After the confinement lady leaves, then that’s when most of the mothers are left on their own and when the fathers go back to work, and they [are] left alone with the baby...[There] will be more things that you [are] worried about, questions they may ask...[but] the people are not around anymore...

T10

#### Subtheme 2: Having a Community of Parents

Mothers from both groups indicated strong preferences for chat groups and interactions with other new parents as it provided communal support, mutual understanding, and encouraged active parenting:

[When] my husband sees more daddies asking questions, he gets more involved. He also reads through and, then, he also asks questions himself

T3

It will be really helpful to have added support from the hospital...because someone who understands you in terms of whatever you say will really make a difference compared to, you know, you speak to your husband and he is not able to understand what you are trying to say.

C2

Let’s say if you are lonely and a struggling mum, you want somebody to listen to you. So, I feel like you need somebody to talk during that crucial time...I thought with this app...somebody is over there, you know, like, watching me, so, okay, I can get through this, you know.

T6

#### Subtheme 3: Enhancement of the Intervention

With regard to the app’s features, 1 mother suggested appointment checking for the baby, such as when vaccinations were due. Another mother suggested a feeding reminder:

When we feed, then we get very busy, then we really forget when the last feed was...When you are passing the baby to another caregiver, then the caregiver doesn’t know when the last time the baby was fed.

C4

A few mothers also reported the need for a segregation and categorization of forum queries so that it would be easier for them to view as well as receive notifications whenever someone replied.

Content-wise, 1 mother suggested adding prenatal information on in vitro fertilization–assisted reproduction to facilitate informed decision making and increase the preparedness of couples undergoing it. She shared her experience:

We underwent the assisted reproduction programme. For the first time, we were quite clueless...because not a lot of people know what it is exactly. So, I think it would be helpful to actually have some sort of guideline to actually know what to expect and so it is not scary...

C4

Although the majority of the mothers in the control group suggested a need for an informational app, most of the mothers in the intervention group requested for extended access to the app for approximately 3 months up to 1 year. Upon termination of the app, 1 mother felt that she was out of it and could not access the app, so it was disappointing to her. Mothers from both the intervention and control groups shared their needs for prolonged use of the app to address different baby issues during the critical first year:

One year is a very crucial time for the baby. I thought I can go into the application, but I realize I do not have the application access. I don’t have enough duration.

T8

If there are queries about the infants at least half a year [postpartum]...I think it will be good if there is some sort of, like, platform or support instead of running to the hospital every single time...An app would be a good option.

C4

The same mother from the control group added:

If it [the app] extended to the prenatal period, it will be good, because, for me, I actually had bleeding in the first trimester, so we were a bit stressed and very scared.

C4

A different mother also suggested the extension of the availability of the app to prenatal stages to increase maternal preparedness before the mother gets too busy with the baby:

Once when the baby comes, then you are...engaged in being with the baby, doing things, you are sleepless also...When you are pregnant, you have more time, so you can go through the educational videos or seek expert advice.

T2

In addition, several mothers from the intervention group requested to have faster responses from the health care professional or to have a live chat as they were usually desperate for answers and the slow responses made them anxious. Overall, 3 mothers shared their experiences:

Our query was answered a bit late...We were really anxious and waiting...So, maybe, you know, this process is faster instead of one person, if [there are] two to three people [replying]. If one is busy, the other one can reply.

T2

When I asked any question, then it takes a day [for it] to be answered. So, if I have some emergency for my baby or anything, I am very much in trouble...

T5

I thought it’s...online when somebody is over there to answer your questions, like instantly...Times like that, my baby was crying, I can’t wait for 24 hours...You are so desperate to get the answers really, really fast...

T6

## Discussion

### Principal Findings

In general, participants in the intervention group were more positive, calm, and well adjusted in the postpartum period. On the other hand, mothers in the control group were most likely to report feeling anxious, lost, and incompetent during the postpartum period. The effectiveness of the technology-based educational intervention in reinforcing parental self-efficacy and improving the emotional well-being of parents during the perinatal stage was similarly reflected in previous studies [24,44-46]. Aligning parents’ expectations with reality and supplying them with parenting information also helped to ease their transition into parenthood [47]. According to Bandura [48], self-efficacy is the optimistic self-belief in our competence or chances of successfully accomplishing a task and producing a favorable outcome. Compared with mothers in the control group, those in the intervention group reported increased parenting confidence and self-efficacy, which also led to other positive parental outcomes such as better parental bonding and improved emotional well-being. This corresponds with previous intervention studies that reported positive intercorrelations between parental outcomes, including social support received [24,49].

With regard to social support, mothers in the intervention group who used the forum felt more reassured and less alone knowing that other parents encountered the same problems. Although sharing with a professional would generate a clinical or scientific reasoning for their physical experiences, sharing with other mothers produced communal responses to the emotional experiences themselves [50]. Therefore, a cohesive community of parents with shared experiences is essential for providing emotional support during the perinatal period [33,34].

The majority of the mothers in this study followed a confinement period and were generally satisfied with the instrumental support received; however, this support was only temporary and delayed mothers’ actual *assumption of duty*, thus causing stress to a minority of the mothers after the confinement period. A plausible reason is the formation of dependence on caregivers during the 1-month confinement period, which hinders the development of parental self-efficacy; however, further studies are needed to verify this claim. Although most studies agree that confinement practices generally improve parental outcomes, it must not be assumed that the confinement period is available to or practiced by all Asians because of the additional financial burden it imposes [22]. Without anyone to turn to, the forum provided a safe space for parents to share their experiences and queries. The anonymity feature of the forum provided a safe haven for mothers to seek answers by reducing the stigma and fear of appearing as incompetent parents [9,51]. Similarly, in other studies [52,53], the anonymity guaranteed by the technology-based intervention greatly reduced help-seeking barriers across the population regardless of medical conditions [54]. In addition, the Chinese superstition of not announcing one’s pregnancy during the first trimester to prevent bad luck serves as a help-seeking barrier for parents in the prenatal stages. This highlights the need for added support by Asian parents in the prenatal period and postconfinement period, which can be met by increasing the duration of accessibility to the app and professional support.

On receiving the intervention, all mothers reported more confidence in their infant care abilities. This increased sense of PSE promoted more immersive parenting, whereby parents were more involved in infant care tasks and actively bonded with their children. Corresponding with Bandura’s social learning theory [48], parental self-efficacy is achieved through mastery experience, verbal persuasion, vicarious experience, and physiological and emotional states. Verbal persuasion from other mothers and the midwife through the app’s forum also served as a form of appraisal support, which motivated mothers to persevere. Being continually assured and reminded that they had the abilities to be proficient at infant care tasks encouraged mothers to work toward achieving better infant care skills [55].

In addition, medical and parenting advice from health care professionals was deemed trustworthy and necessary by parents to ease their worries and anxiety in the perinatal period. Mothers who lacked professional advice reported poorer self-esteem and emotional well-being, which negatively influenced mothers’ sense of self-efficacy. This is congruent to the emotional aspect of Bandura’s learning theory [48], whereby a poor emotional or physical state adversely affects PSE. Therefore, receiving guidance from a health care professional is a vital facet to parents developing a sense of security during the postnatal period [24,56].

Various features of the intervention also enhanced mothers’ vicarious experiences, which boosted their confidence and self-efficacy. First, the forum established a platform for parents to post queries, share experiences, and provide advice that was moderated by the midwife. Mothers were better equipped with parenting skills through the observation and imitation of other parents’ techniques. In addition, there was increased paternal involvement when fathers noticed other fathers’ active involvement in the forum. The imitation bias of mimicking a similar individual increased the self-relevance and adaptive value of the information learned [48,57]. A study further discovered that own-gender imitation behavior actually elicits a reward response in the brain [58]. This emphasizes the need for the extension of technology-based supportive community networks to encourage active and immersive parenting.

In consideration of parental preferences for the dissemination of information through multimedia [59], the audio and video recordings on baby care skills proved a useful reference guide for parents to model after. Studies have also reported higher memory retention through visual imagery than auditory or written contents [60], which suggests the effectiveness of the app in boosting parental learning and retention compared with didactic perinatal lectures provided by the hospital. Mothers also recommended a categorization of information on the app for ease of reference and learning. Likewise, Bandura stated that organized information better enhances memory than fragmented information [48].

However, the technology-based SEPP failed to incorporate the mastery experience facet of Bandura’s social learning theory [48] as only mothers who had given birth previously could achieve mastery experience. The technology-based intervention did not provide a direct mastery of infant and self-care skills, which is equally vital for promoting self-efficacy. In Saunders et al’s study [61], mothers who received hands-on practice improved faster and better incorporated those strategies into their parenting behaviors. Therefore, there is still a necessity for hands-on practice and positive live guidance from health care professionals, suggesting that technology-based interventions should be used as a supplementary tool to existing hospital care and not as a replacement [62].

Overall, the SEPP was generally well received, with certain feedback regarding the expansion of content database and information coverage (eg, in vitro fertilization) as well as the app’s availability during the antenatal period. With such improvements, the SEPP can potentially address the common issue of the lack of informed decision making during the antenatal period [63]. Suggestions to include features such as appointment reminders and the categorization of forum queries will also enhance user friendliness and increase usability, creating an all-in-one go-to app for parents. In many studies [24,64], asynchronous communication with health care professionals was reported to be of utmost importance to mothers who see such apps as a lifeline. Although our study has adopted a similar approach through the Web-based forum, mothers gave feedback that the responses from the midwife were too slow, which was not reported in Danjborg et al’s study [24], which used Web-based chat instead. Therefore, future studies can consider including an instant messaging function by using an Web-based chat to contact health care professionals directly.

### Implication for Future Research and Clinical Practice

Given the rapid advancement of technology and the various benefits technology provides, such as cost-effectiveness, flexibility, accessibility, and its potentially extensive outreach to the wider community, this should serve as an impetus for health care sectors to dedicate more resources to further develop such technology-based interventions.

In a collectivist Asian society that strongly places emphasis on family-oriented values, one’s family is often the immediate source of emotional support, and the lack of it may adversely affect a mother’s emotional well-being [65]. Along with the rise of paternal involvement in parenting, future interventions should tailor features to encourage involvement and support from other family members such as grandparents. With an increasing dependency on Web-based communities for support, future studies should also emphasize on building more interactive or live communities for parents to interact and exchange experiences.

### Strengths and Limitations

Unique to this study is the added insight on Asian cultures such as taboos and traditions during the perinatal period. To the best of our knowledge, this is also the first qualitative study to provide in-depth insight into participants’ views on a multimodal technology-based SEPP that was implemented during the perinatal period. A major limitation of this study was the unavailability of fathers during the follow-up interviews, which impeded our understanding of paternal experiences and perceptions of the SEPP intervention. However, mothers who were interviewed mostly reported positive receptivity by fathers and increased fathers’ involvement in infant care. The once-off nature of this study disallowed the comparison of pre- and posttest perceptions of mothers, making it difficult to determine the effectiveness of the intervention. In addition, using the convenience sampling method and voluntary participation did not provide a good representation of mothers in the control group. Therefore, future studies should consider employing random or purposive sampling methods when recruiting participants for interviews. The mobile app was a novel approach and the last intervention phase of the SEPP; hence, because of possible recency effect, participants’ responses were more inclined to the mobile app. Future qualitative studies should, therefore, consider constructing component-specific interview questions to have a more holistic evaluation of the entire intervention program.

### Conclusions

This study examined the perceptions of participants who received routine care and those who received the technology-based SEPP intervention in addition to routine care in the perinatal period. A comparison of mothers’ perceptions in both the groups allowed a deeper insight into the needs of mothers in the perinatal period, with the intervention group being generally satisfied with the SEPP. The multifeature technology-based intervention was effective in improving parental outcomes and was well received by parents. This calls for a higher allocation of resources to further develop and tailor technology-based interventions that can supplement existing hospital perinatal care and be implemented in a wider community of parents.

## Abbreviations

C-sect: cesarean section
mHealth: mobile health
NHG DSRB: National Health Group Domain Specific Review Board
NS: not stated
NVD: normal vaginal delivery
PSE: parenting self-efficacy
RCT: randomized controlled trial
SEPP: supportive educational parenting program

## Appendix

Multimedia Appendix 1 COREQ Checklist.
